# Large Extracellular Vesicles Derived from Natural Killer Cells Affect the Functions of Monocytes

**DOI:** 10.3390/ijms25179478

**Published:** 2024-08-31

**Authors:** Dmitry Sokolov, Alina Gorshkova, Elizaveta Tyshchuk, Polina Grebenkina, Maria Zementova, Igor Kogan, Areg Totolian

**Affiliations:** 1 Federal State Budgetary Scientific Institution “The Research Institute of Obstetrics, Gynecology and Reproductology Named after D.O. Ott”, 199034 St. Petersburg, Russia; 2 Saint-Petersburg Pasteur Institute, 197101 St. Petersburg, Russia

**Keywords:** natural killers, monocytes, large extracellular vesicles, ectosomes, NK-92, THP-1, PBMC

## Abstract

Communication between natural killer cells (NK cells) and monocytes/macrophages may play an important role in immunomodulation and regulation of inflammatory processes. The aim of this research was to investigate the impact of NK cell-derived large extracellular vesicles on monocyte function because this field is understudied. We studied how NK-cell derived large extracellular vesicles impact on THP-1 cells characteristics after coculturing: phenotype, functions were observed with flow cytometry. In this study, we demonstrated the ability of large extracellular vesicles produced by NK cells to integrate into the membranes of THP-1 cells and influence the viability, phenotype, and functional characteristics of the cells. The results obtained demonstrate the ability of large extracellular vesicles to act as an additional component in the immunomodulatory activity of NK cells in relation to monocytes.

## 1. Introduction

Monocytes, macrophages, and NK cells are essential components of the innate immune system. NK cells are CD3-negative large granular lymphocytes of the innate immune system that make up 10–15% of all circulating lymphocytes in human blood [1,2]. Monocytes are myeloid cells that circulate in blood for up to three days before migrating to tissues, where they differentiate into macrophages in response to specific environmental factors [3].

During the immune response, cells interact with each other in a specific manner, creating a unique microenvironment that either activates or inhibits the activity of other cells [4,5]. This interaction is primarily achieved through direct intercellular contact or by the activation of various biologically active molecules, primarily cytokines [4,6].

NK cells are able to influence the differentiation of monocytes in various ways. For example, in rheumatoid arthritis, CD56^bright^ CD16^dim/−^ NK cells have been shown to contribute to the differentiation of monocytes into osteoclasts, which leads to increased bone destruction in this condition [7]. In addition, these same cells in lymph nodes and inflamed tissues have been found to direct the differentiation of monocytes into dendritic cells by secreting GM-CSF, a cytokine that promotes this process. The secretion of GM-CSF is enhanced via the activation of NK cells through the action of IL-15. IL-15 is produced in various cell types, including monocytes [8]. This mutual activation can also occur through the interaction between the NKp80 receptor on NK cells and the activation-induced C-type lectin CLEC2B on monocytes. This interaction leads to an increased production of TNFα in both cell types, as well as increased expression of IFNγ via NK cells [9].

NK cells are able to destroy hyperactivated macrophages [10]. In response to the excessive activation of macrophages, the expression of ligands for NKG2D receptors on NK cells, MICA and MICB, increases on the surface of NK cells. This leads to ligand-receptor interaction between NK cells and macrophages, resulting in the lysis of the macrophages. This mechanism is likely aimed at eliminating overstimulated macrophages to prevent endotoxic shock [11]. The lack of NK cell regulation of macrophages can lead to inflammation and various pathological conditions [10].

It has been shown that IFNγ, produced via NK cells, regulates the polarization of macrophages, directing them towards the pro-inflammatory (M1) phenotype [12,13]. However, other NK cell-derived factors (GM-CSF, TNFα, and MIP1αß) can also activate macrophages [5,14]. It was also found that the number of macrophages in an inflammatory focus is dependent on the number of NK cells present, with a decrease in NK cell numbers leading to a reduction in macrophage and neutrophil infiltration, as well as a shift in macrophage polarization towards the M2 phenotype [15].

Cells release membrane vesicles of various types into the extracellular space with a membrane that both has an endosomal origin (exosomes) and forms from the plasma outer membrane of the cell (ectosomes) or apoptotic bodies [16,17,18]. The diameter of the exosomes is up to 100 nm, the diameter of the ectosomes ranges from 100 to 1000 nm [19], and the diameter of apoptotic bodies is above 1000 nm. The methods of the formation, composition, and functions of all of these vesicles are different. There are no recognized markers specific for ectosomes that are not present on exosomes. For example, CD63, and CD9 were considered to confirm that the isolated extracellular vesicles were exosomes [20]. However, Kowal J. et al., 2016 [21] showed that CD63 and CD9, previously considered to be specific exosome markers, were present on extracellular vesicles obtained after centrifugation at 2000× *g* (apoptotic bodies), 10,000× *g* (apoptotic bodies and ectosomes), and 20,000× *g* (ectosomes). Therefore, at present, one of the most adequate ways to obtain a pure ectosome fraction is the method of differential centrifugation [22,23]. In accordance with the Minimal Information for Studies of Extracellular Vesicles (MISEV2023) guidelines [18], “ectosomes” can only be used as proof for subcellular origin extracellular vesicles (EVs). In the absence of a clear-cut body of evidence, at present, the MISEV2023 guidelines delineate the categorization of such entities based on their compositional makeup, dimensions, and other attributes. According to the MISEV2023 guidelines, the dimensions of the resulting particulates may fluctuate during the course of differential centrifugation procedures. Based on the diameter of the separated particles, Small EVs (SEVs) are often described as being <200 nm in diameter and large EVs (LEVs) are often described as being >200 nm in diameter.

Currently, it is hypothesized that extracellular vesicles, including ectosomes, may function as an additional method of communication between cells [24]. Ectosomes are small, membranous particles that are released via different types of cells, including NK cells. It has been demonstrated that ectosomes contain various intracellular molecules from the source cell, as well as receptors, adhesion molecules, and other proteins [25,26]. The presence of these diverse biologically active substances in ectosomes, along with the wide range of receptors on their surface suggests that these particles may play a role in various physiological and pathological processes within the body, such as inflammation and the immune response [27,28,29].

Due to the fact that NK cells are a small population of leukocytes, there is a lack of data on the characteristics and functions of their ectosomes\LEVs. However, it has been established that NK cells can secrete extracellular vesicles, including ectosomes. It has been demonstrated that molecules specific to the source cells are expressed both within and on the surface of NK cell-derived ectosomes [30]. This suggests that the effects of NK cell-derived ectosomes and the NK cells themselves on target cells may be similar [31,32]. In particular, the ability of NK cells to produce ectosomes in order to enhance the expression of costimulatory molecules such as CD80 and CD86 on the surface of peripheral blood monocytes has been shown [33]. To date, there is a lack of data on the effect of EVs produced via NK cells, and especially ectosomes, on macrophages. However, it is assumed that this mechanism of communication between NK cells and monocytes/macrophages may play an important role in immunomodulation and the regulation of inflammatory processes.

Until now, the overall understanding of the interactions between NK cells and monocytes/macrophages has failed to consider the potential for these cells to communicate with each other through ectosomes. Consequently, the aim of this research was to investigate the impact of NK cell-derived ectosomes\LEVs on monocyte function.

## 2. Results

### 2.1. A High Concentration of LEVs Derived from NK Cells Causes a Decrease in the Viability of THP-1 Cells

**Figure 1:**
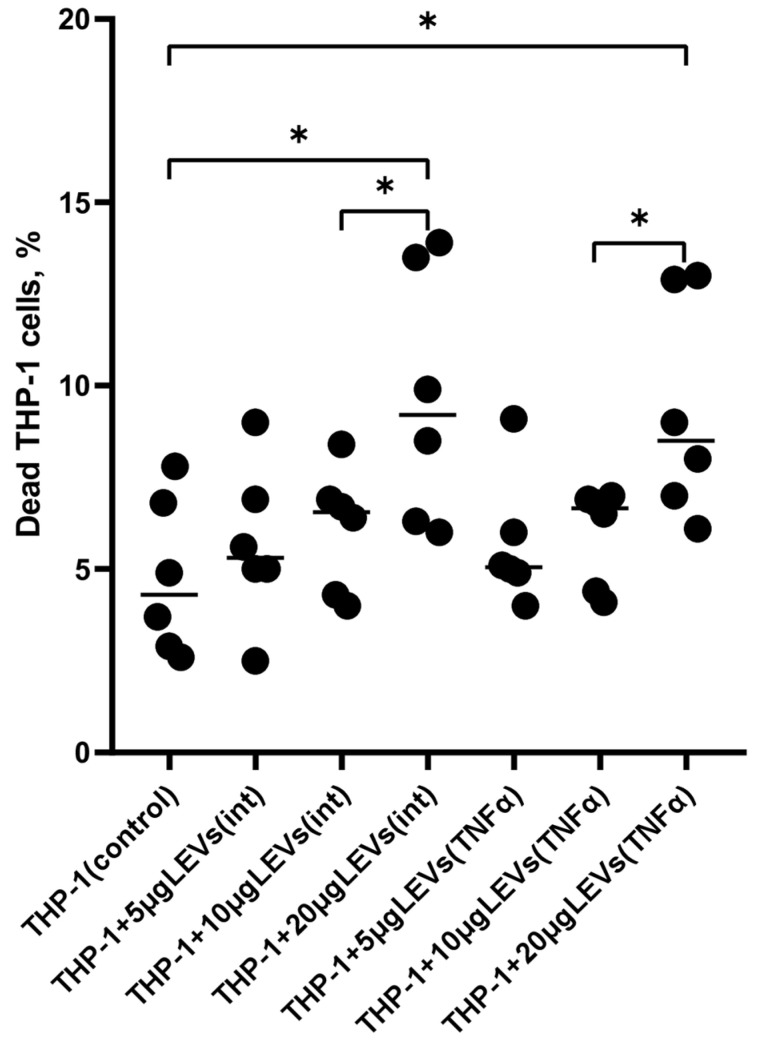
The percentage of dead THP-1 cells after their co-culturing with LEVs derived from NK-92 cells. THP-1(control)–THP-1 cells stained with 7-AAD; 5 μg/10 μg/20 μg LEVs (int)–THP-1 cells stained with 7-AAD after a co-culture with LEVs(int) at three different concentrations (5, 10, or 20 μg of protein per 100 μL); and 5 μg/10 μg/20 μg LEVs(TNFα)—THP-1 cells stained with 7-AAD after a co-culture with LEVs(TNFα) at three different concentrations (5, 10, or 20 μg of protein per 100 μL). Significant differences: *—*p* < 0.05.

In this study, we used TNFα as an inductor since previously the analysis of the cytotoxic activity of NK-92 cells in an in vitro NK-cell assay using K562 target cells had demonstrated that LEVs derived from TNFα-stimulated NK-92 cells increased the cytotoxicity of those cells. This coincides with the previous finding of an increased content of granzyme B in LEVs derived from TNFα-stimulated cells of the NK-92 line. This is described in more detail in our paper [28]. First, we tested different concentrations of LEVs (5, 10, or 20 μg of protein per 100 μL) and found that only the highest concentration of LEVs (20 μg/100 μL) derived from NK-92 cells was a decrease in the viability of THP-1 cells caused (Figure 1, Supplementary Data, Table S2). In order to avoid a cytotoxic effect on THP-1 cells, all further experiments were carried out using a LEVs concentration of 10 µg.

### 2.2. LEVs Derived from CFSE-Stained NK Cells Transfer a Fluorescent Label to THP-1 Cells

**Figure 2:**
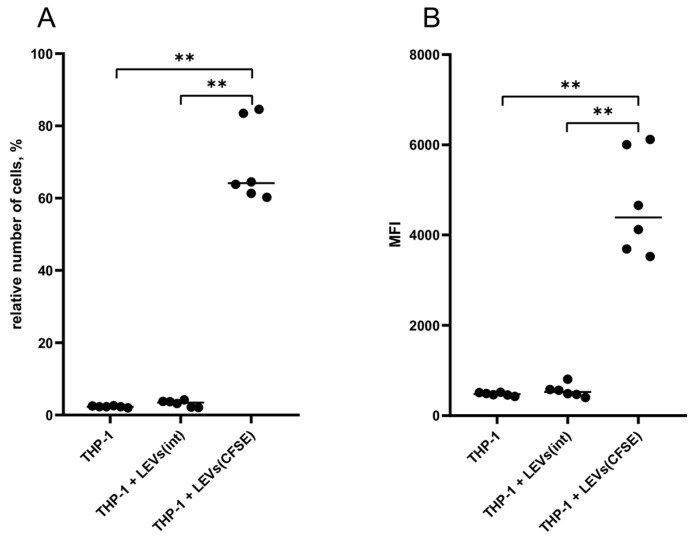
The percentage (**A**) and mean fluorescence intensity (MFI) (**B**) of THP-1 cells, incubated with LEVs derived from NK-92 cells. THP-1 intact cells; THP-1 + LEVs(int)—THP-1 cells cultivated in the presence of LEVs(int); and THP-1 + LEVs(CFSE)—THP-1 cells cultivated in the presence of LEVs(CFSE). Significant differences: **—*p* < 0.01.

THP-1 cells were cultured in the presence of LEVs derived from CFSE-treated NK-92 cells (LEVs (CFSE)). As a result, there was an increase in the level of fluorescence of THP-1 cells compared to the autofluorescence level of intact THP-1 cells (negative control) and the fluorescence level of THP-1 cells cultured with LEVs derived from intact NK-92 cells (LEVs(int)) (Figure 2, Supplementary Data, Table S1).

### 2.3. TNFα and LEVs Derived from TNFα-Activated NK Cells Increase the Phagocytic Activity and the Oxidative Burst of THP-1 Cells

**Figure 3:**
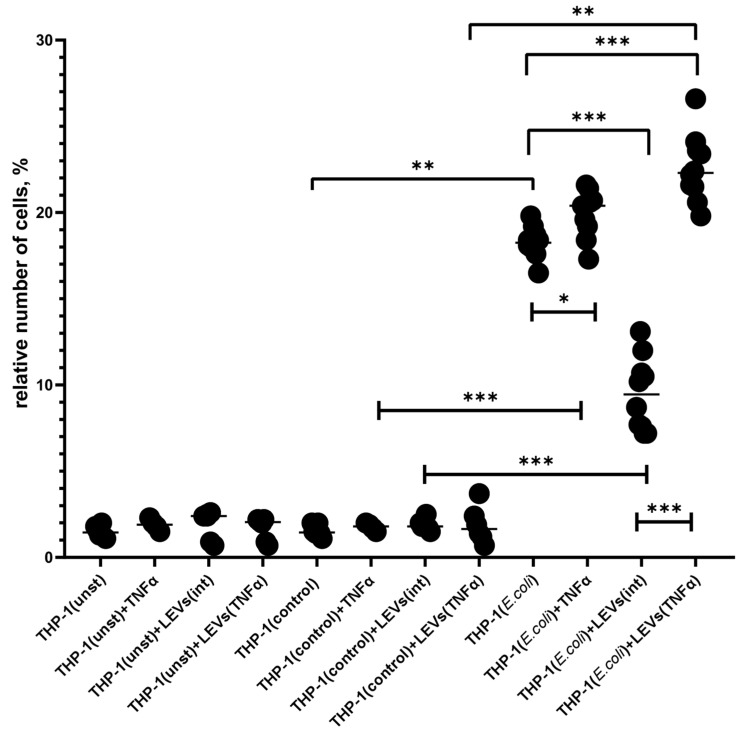
The percentage of FITC-positive THP-1 cells after the phagocytosis of FITC-labeled *E. coli.* THP-1(unst)—intact THP-1 cells; THP-1(control)—THP-1 cells stained with tripan blue (TB) and propidium iodide (PI); THP-1(*E. coli*)—THP-1 cells stained with TB and PI after incubation with FITC-labeled *E. coli*; +TNFα—THP-1 cells pre-incubated with TNFα; +LEVs(int)—THP-1 cells pre-incubated with LEVs(int); and +LEVs(TNFα)—THP-1 cells pre-incubated with LEVs(TNFα). Significant differences: *—*p* < 0.05; **—*p* < 0.01; and ***—*p* < 0.001.

We demonstrated that the activation of THP-1 cells with TNFα leads to an increase in their phagocytic capacity, as compared to the level of phagocytosis of intact THP-1 cells (Figure 3). Prior incubation of THP-1 cells with LEVs(int) caused a decrease in phagocytic activity compared to the spontaneous level (Figure 3). However, the incubation of THP-1 cells with LEVs produced via TNFα-activated NK-92 cells (LEVs(TNFα)) resulted in an increased phagocytic activity compared to both the control group and the level shown for cells incubated with LEVs(int) (Figure 3, Supplementary Data, Table S3).

**Figure 4:**
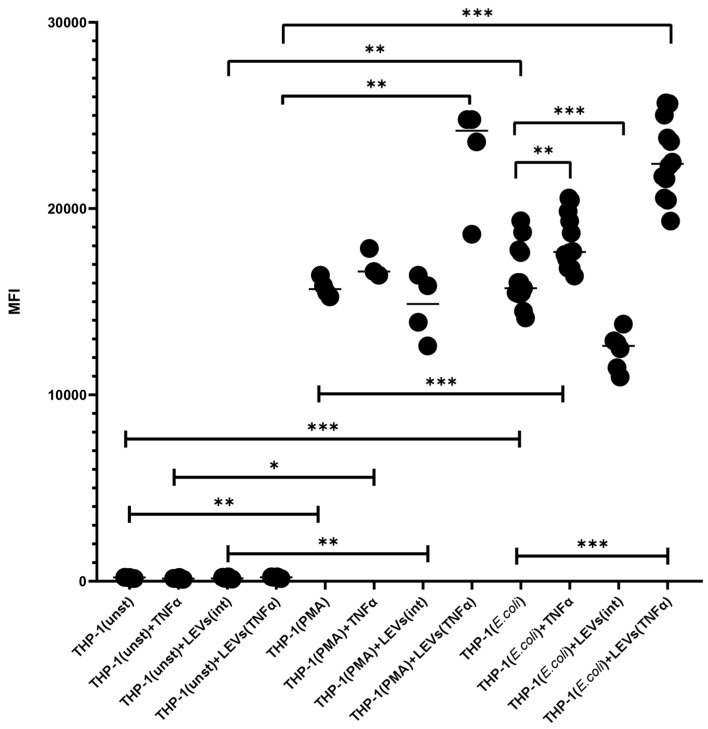
The mean fluorescent intensity (MFI) of THP-1 cells after the phagocytosis of unstained *E. coli* in the presence of dihydrorhodamine 123. THP-1(unst)—intact THP-1 cells; THP-1(PMA)—THP-1 cells treated with phorbol-12-myristate-13-acetate (PMA); THP(*E. coli*)—THP-1 cells that have undergone oxidative explosion after activation with FITC-labeled *E. coli*; +TNFα—THP-1 cells pre-incubated with TNFα; +LEVs(int)—THP-1 cells pre-incubated with LEVs(int); and +LEVs(TNFα)—THP-1 cells pre-incubated with LEVs(TNFα). Significant differences: *—*p* < 0.05; **—*p* < 0.01; and ***—*p* < 0.001.

The activation of THP-1 cells with TNFα also resulted in an increased oxidative burst compared to the level seen in intact THP-1 cells (Figure 4, Supplementary Data, Table S4). The pretreatment of THP-1 cells with LEVs(int) decreased the level of the oxidative burst in THP-1 cells compared to the level seen in intact THP-1 cells. At the same time, the treatment of THP-1 cells with LEVs(TNFα) enhanced the intensity of the oxidative burst in THP-1 cells compared to the level seen in intact THP-1 cells and also compared to the level in THP-1 cells pre-treated with LEVs(int) (Figure 4).

### 2.4. LEVs Derived from NK-92 Cells Alter the Phenotype of THP-1 Cells

**Figure 5:**
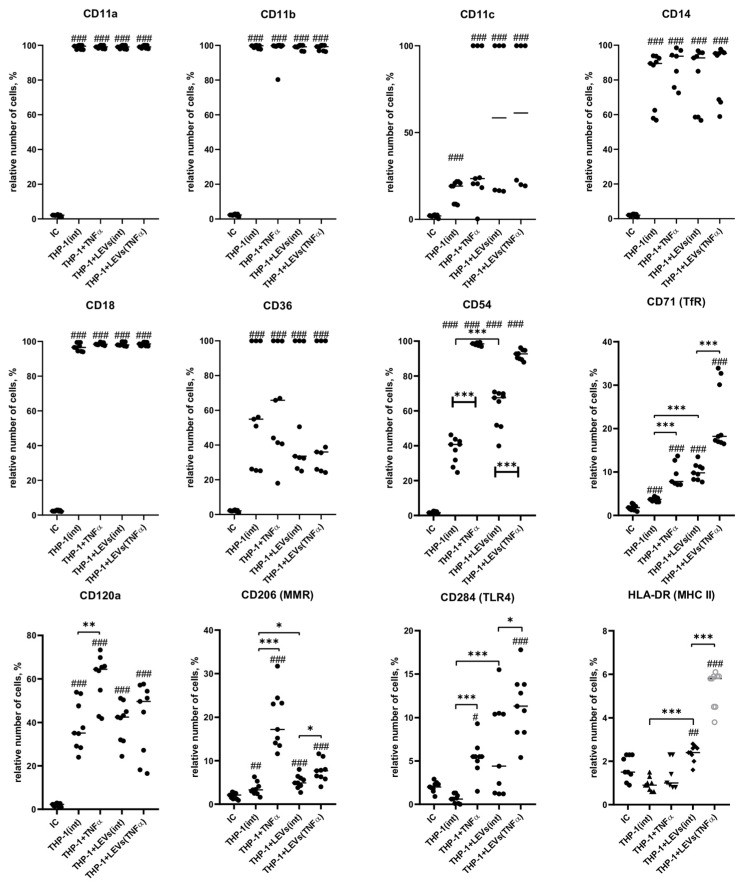
The percentage of THP-1 cells expressing receptors in the presence of LEVs(int) or LEVs(TNFα). IC—isotypic control; THP(int)—intact THP-1 cells; THP + TNFα—THP-1 cells pre-incubated with TNFα; THP + LEVs(int)—THP-1 cells pre-incubated with LEVs(int); and THP + LEVs(TNFα)—THP-1 cells pre-incubated with LEVs(TNFα). Significant differences: *—*p* < 0.05; **—*p* < 0.01; ***—*p* < 0.001; #–the difference with isotypic control; #—*p* < 0.05; ##—*p* < 0.01; and ###—*p* < 0.001.

**Figure 6:**
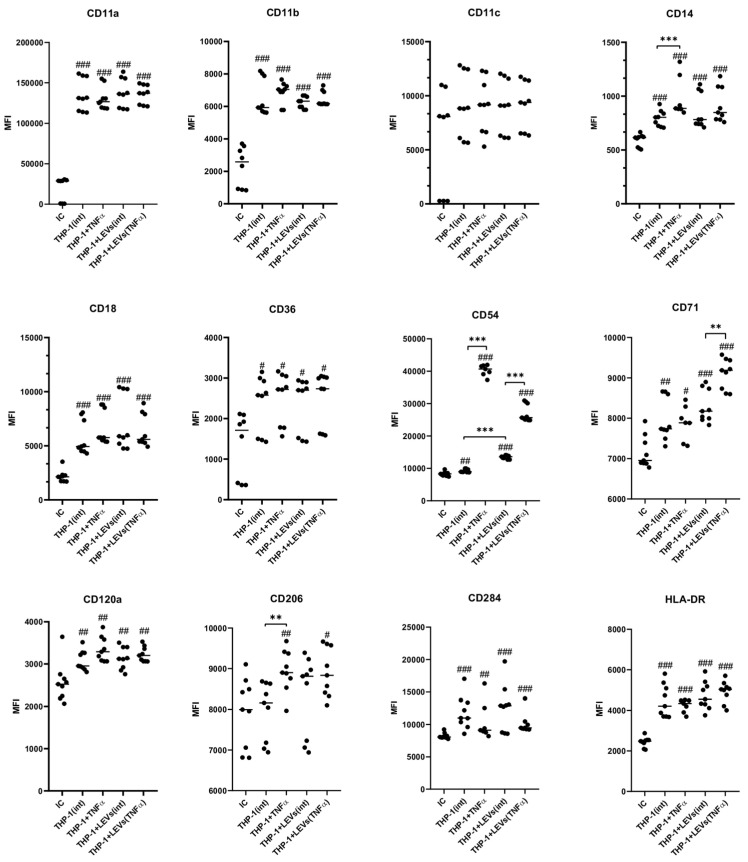
The mean fluorescence intensity (MFI) (B) of THP-1 cells expressing receptors in the presence of LEVs(int) or LEVs(TNFα). IC—isotypic control; THP(int)—intact THP-1 cells; THP + TNFα—THP-1 cells pre-incubated with TNFα; THP + LEVs(int)—THP-1 cells pre-incubated with LEVs(int); and THP + LEVs(TNFα)—THP-1 cells pre-incubated with LEVs(TNFα). Significant differences: **—*p* < 0.01; ***—*p* < 0.001; #–the difference with isotypic control; #—*p* < 0.05; ##—*p* < 0.01; and ###—*p* < 0.001.

It has been determined that intact THP-1 cells express the following surface molecules: CD54, CD71, CD206, CD120a, CD14, CD36, CD11a, -b and -c, and CD18. The expression of HLA-DR and CD284 in unstimulated cells was not detected (Figure 5 and Figure 6). The stimulation of THP-1 cells with TNFα led to an increased expression of CD284 as well as a higher number of cells expressing CD54, CD71, and CD206 compared to unstimulated cells (Figure 5). Additionally, the stimulation also enhanced the expression intensity of CD54 and CD206 receptors (Figure 6). A co-culture of THP-1 cells with LEVs(int) resulted in an upregulation of HLA-DR expression (Figure 5 and Figure 6) and an increase in the number of CD54+ and CD71+ cells (Figure 5). Additionally, there was an increase in the intensity of CD54 expression (Figure 6) when compared to the control. A co-culture of THP-1 cells with LEVs(TNFα) led to an increase in the number of cells expressing HLA-DR, CD284, CD206, CD54, and CD71, compared to the number observed co-cultured with LEVs(int). In addition, a higher expression intensity of CD54 and CD71 was seen when THP-1 cells were co-cultured with LEVs(TNFα), compared to the level shown when co-cultured with LEVs(int) (Figure 6). Different cultivation conditions did not affect the number of THP-1 cells expressing CD14, CD36, CD18, CD11a, CD11b, and CD11c (Figure 5, Supplementary Data, Table S5), or the intensity of expression of CD120a, CD284, CD36, CD18, CD11a, CD11b, and CD11c (Figure 6, Supplementary Data, Table S6).

### 2.5. LEVs Derived from NK-92 Cells Decrease the Phagocytic Activity and the Oxidative Burst of PBMCs

**Figure 7:**
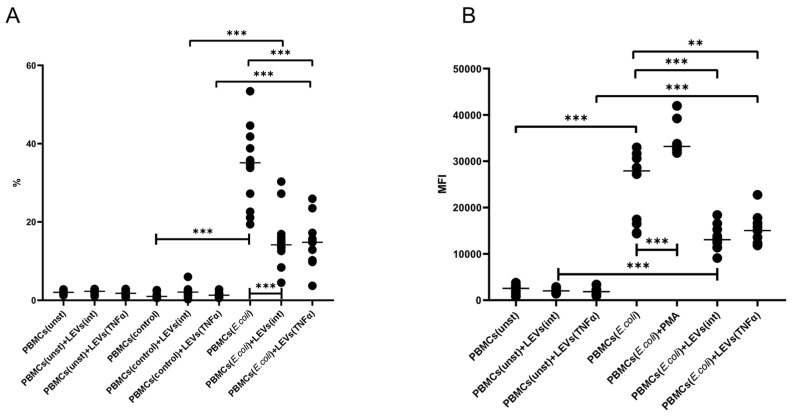
The percentage of FITC-positive PBMCs after the phagocytosis of FITC-labeled *E.coli* (**A**) and the mean fluorescent intensity (MFI) of PBMCs after the phagocytosis of unstained *E. coli* in the presence of dihydrorhodamine 123 (**B**) in the presence of LEVs(int) or LEVs(TNFα). PBMC(unst)–oxidative burst of intact PBMCs; PBMC(*E. coli*)—baseline level of oxidative burst of PBMCs stimulated by FITC-labeled *E. coli*; +PMA—oxidative burst in the presence of PMA (phorbol-12-myristate-13-acetate); and +LEVs—PBMCs treated with LEVs(int); +LEVs(TNFα)—PBMCs treated with LEVs(TNFα). Significant differences: **—*p* < 0.01; ***—*p* < 0.001.

It was found that the cultivation of PBMCs with LEVs(int) or LEVs(TNFα) resulted in a decreased phagocytic capacity of PBMCs and reduced intensity of oxidative burst, compared to baseline levels (Figure 7, and Supplementary Data, Tables S7 and S8).

## 3. Discussion

Currently, the role of different types of extracellular vesicles in facilitating intercellular communication is a topic of active research [24]. It has been shown that almost all cells of the body, including NK cells [34], are capable of producing EVs [35]. In this study we investigated the impact of NK cells on the functional status and phenotypic characteristics of monocyte-like THP-1 cells. NK-92 cells were chosen as a source of EVs, as they exhibit the phenotypic and functional features of NK cells [36].

Due to their ability to transport biologically active molecules, ectosomes\LEVs are considered to be subcellular messengers that transmit signals to surrounding cells [28,37]. The contact of ectosomes\LEVs with the recipient cells leads to signal transmission and occurs through both ligand-receptor interactions and the release of ectosomes\LEVs content into the extracellular space [38]. In addition, ectosomes\LEVs are able to enter the cells via endocytosis, and fuse with the plasma membrane of recipient cells and transfer their contents directly into the cytosol [38]. These mechanisms allow ectosomes\LEVs to trigger various intracellular signaling pathways, altering the functional and phenotypic characteristics of recipient cells [39]. We found that the incubation of THP-1 cells with a medium containing LEVs derived from fluorescently labeled NK-92 cells leads to the transfer of an intracellular protein labeled with a fluorescent dye to monocyte-like cells. These findings are consistent with the results of numerous researchers who have demonstrated the ability of EVs, including ectosomes\LEVs, from various cell types, to internalize within recipient cells [40,41,42].

To assess the functional capabilities of LEVs derived from NK cells in relation to monocyte-like cells, we investigated the effect of NK-92-derived LEVs on the viability of THP-1 cells. We found that the highest tested concentration of LEVs (20 μg/100 μL) was able to reduce the viability of approximately 15% of THP-1 cells. This result could be attributed to the presence of perforin and granzyme B in ectosomes\LEVs [28,43]. These proteins, which are also part of the lytic granules of NK cells, together with granulysin, FasL, and TRAIL, are released upon contact with a target cell causing its apoptosis [44]. It is possible that in our study high concentrations of LEVs could have caused a decrease in cell viability due to the apoptosis of THP-1 cells. The results we obtained regarding the reduction in the viability of monocyte-like cells and the cytotoxic activity of LEVs produced via NK cells, as described previously [28], which may support the non-classical theory of cell lysis [28]. At present, it is not entirely clear how granzyme B is transported to target cells. In addition to the well-established theory of cellular cytolysis, which states that perforin forms pores in the cell membrane, allowing granzyme B to enter the cytoplasm [44], there is another theory that suggests that LEVs act as carriers for perforin and granzyme B within target cells [31]. Perforin then forms pores in the endosomal membrane, and granzyme B enters the cytoplasm, initiating apoptosis [43]. Therefore, the presence of lytic proteins in NK cells [28] suggests that they may constitute a novel mechanism through which these cells are able to exert their cytotoxic function, including in relation to monocytes. In the absence of inflammation and stimulation via factors such as LPS, TNFα, and IL-1β, monocytes perceive apoptotic signals from surrounding cells, including NK cells, and are eliminated from tissues [45,46]. In this context, the observed reduction in the viability of THP-1 cells caused by LEVs derived from NK cells can be considered an additional mechanism for immunoregulating the monocyte function by NK cells, as well as for maintaining the number of tissue-resident monocytes available for immune responses.

The primary function of monocytes is to provide innate immunity, which protects against pathogens and maintains tissue homeostasis [47]. The regulation of monocyte activity is provided via various cells of the innate and adaptive immune systems [48,49]. Specific interactions between monocytes and these cells occur through ligand-receptor communication or through the secretion of biologically active substances. These interactions result in the establishment of a unique microenvironment that defines the outcome of inflammation [5]. Currently, the role of various types of EVs in the process of communication during inflammation is being actively investigated. However, there is a lack of data on the effect of LEVs derived from NK cells on monocytes. Therefore, we set out to analyze the potential of NK cells to modulate the phenotypic and functional characteristics of monocytes through LEVs.

The ability of LEVs produced via NK cells to alter the phenotypic features of monocytes was assessed through the analysis of the expression of various functional receptors (see the table in Section 4). THP-1 cells, which reflect the characteristics of promonocytic cells, are able to differentiate into various populations of monocytes and macrophages when subjected to different inducers [50,51]. The results obtained from this study regarding the spontaneous expression of molecules via intact THP-1 cells align with previously published data on their phenotypic characteristics. In particular, more than half of the cells expressed CD14, which is a classic marker for monocytes [50,52], as well as TNF receptor (CD120a) [53] and integrins CD11a, CD11b, and CD18 [54]. At the same time, these cells exhibited a weak expression of CD71, CD206, and CD54 molecules [54,55,56], and they also did not express HLA-DR [57,58] and CD284 molecules [59]. This indicates their undifferentiated state and is consistent with the literature. The expression of many surface markers on THP-1 cells can be triggered via treatment with PMA, LPS, or cytokines [50,51]. To simulate the conditions of monocyte activation during the immune response, THP-1 cells were activated with the pro-inflammatory cytokine TNFα. As expected, we observed an increased level of the expression of the CD54, CD71, and CD206 molecules, which are characteristic of activated monocytes. Additionally, the number of THP-1 cells expressing CD120a and CD284 increased, consistent with previous reports on the inducibility of these surface markers in THP-1 cells [53,54,55,56,59]. The activation of THP-1 cells by TNFα apparently induced the differentiation of these cells into mature monocytes, as evidenced by the results of the functional tests.

After that, we assessed the ability of LEVs produced via NK cells to alter the phenotypic features of THP-1 cells. To accomplish this, we cultured THP-1 cells with LEVs derived from intact or TNFα-activated NK-92. The activation with TNFα was used in order to simulate the inflammatory activation of NK cells [1]. It was found that the co-culture with LEVs derived from intact NK cells resulted in the emergence of THP-1 cells expressing HLA-DR, as well as an increase in the number of cells expressing CD54, CD71, and CD206. The co-culture with LEVs derived from the TNFα-stimulated NK cell led to the emergence of CD284+ cells and an even higher increase in the expression of HLA-DR, CD284, CD206, CD54, and CD71 relative to the levels observed in the presence of LEVs(int). These results are likely connected to the varied internal and surface molecules of LEVs, which can be transmitted to target cells through fusion or endocytosis [27,33,38]. Although the exact mechanisms of the interaction between monocyte cells and LEVs have not yet been determined, we found that LEVs derived from NK cells are able to alter the phenotypic characteristics of THP-1 cells. Furthermore, the observed effect is similar to that seen when THP-1 themselves are activated via TNFα. As previously stated, CD54, CD71, CD206, and CD120a are inducible markers whose expression increases on monocytes upon activation and polarization via various cytokines and activating agents [53,54,55,56,59]. In addition to cytotoxic proteins, numerous proinflammatory and immunomodulatory molecules have been found in LEVs derived from NK cells: interferon-gamma (IFN-γ), interleukin-1 beta (IL-1β), and tumor necrosis factor alpha (TNFα) [60], as well as various microRNAs [61]. The evidence for the presence of these cytokines in LEVs is supported by the appearance of human leukocyte antigen HLA-DR expression on the surface of THP-1 cells after cultivation with LEVs, but not with TNFα. According to the literature, HLA-DR is not expressed through unstimulated THP-1 cells, however, its expression is observed when the cells are stimulated via cytokines [57,58]. Furthermore, HLA-DR expression can only be induced in the presence of both TNFα and IFNγ [58]. Therefore, the induction of HLA-DR on monocyte-like cells appears to be associated with the presence of IFNγ and TNFα in the LEVs derived from NK cells. It has been suggested that the ability of NK cells to generate vesicles with different internal compositions, depending on their activation status, ref. [33] may explain the variation in the level of expression of other markers observed on THP-1 cells. It should also be noted that LEVs and receptors on their surfaces can be incorporated into the membranes of target cells [42]. Consequently, the increased expression of the CD284 receptor on THP-1 cells after their incubation with LEVs may be linked to the integration of the membranes of LEVs derived from NK-92 cells, which are known to express CD284 (see table in Section 4.8).

The ability of NK cells to influence the phagocytic activity and the oxidative burst of monocytes has been evaluated through the analysis of the interaction between THP-1 cells and FITC-labeled *E. coli* after the pre-incubation of cells with LEVs derived from intact or TNFα-activated NK cells. The results obtained for the baseline levels of phagocytosis and oxidative burst in intact THP-1 cells are consistent with the literature’s data [50,51]. The increased phagocytic activity and oxidative response induced via TNFα-activated THP-1 cells also corresponds to the classical theory of pro-inflammatory monocyte activation [5,14,62]. The increased functional activity of TNFα-activated THP-1 cells is likely related to an increase in the number of cells expressing the mannose receptor, CD206, on their surface. This protein plays an important role in facilitating the initial stages of phagocytosis by facilitating the uptake of bacterial cell wall components [56,63].

Pre-incubation of THP-1 cells with LEVs derived from intact NK-92 cells led to a decrease in the intensity of phagocytosis by THP-1 cells and a reduction in the production of reactive oxygen species (ROS) compared to the spontaneous levels. This effect was also observed in PBMCs from healthy donors. These findings suggest that LEVs produced via NK-92 cells can transmit inhibitory signals to phagocytes, potentially leading to anti-inflammatory responses. These effects can be mediated through various substances transported in vesicles, specifically anti-inflammatory cytokines (IL-10 and TGFβ) or microRNAs, that affect the expression of genes in target cells [61]. According to the literature data, EVs derived from NK cells can influence various signaling pathways in target cells, although the mechanisms underlying this interaction are not fully understood. Specifically, NK cell-derived exosomes have been shown to influence the activation of phosphatidylinositol-3 kinase (PI3K) [60], which plays a crucial role in activation of actin cytoskeletal rearrangements and the formation of phagocytic cup upon contact with pathogens [64]. A reduction in the intensity of oxidative burst is a direct result of a reduction in phagocytic activity, as the assembly of NADPH oxidase complexes, which produce ROS, occurs on membranes formed via phagolysosomes [64,65].

The pre-treatment of THP-1 cells with LEVs produced through TNFα-activated NK-92 cells resulted in increased phagocytic activity and oxidative burst, compared to both the spontaneous levels and the levels shown in TNFα-activated THP-1 cells. These findings are consistent with the observed changes in the phenotypical properties of the cells. Therefore, the activation of THP-1 cells via the components or surface molecules of LEVs affects the phenotypic features of the cells and directly influences their functional abilities. It is noteworthy that, despite the fact that the functional outcomes observed during the incubation of THP-1 cells with TNFα or LEVs produced via TNFα-activated NK cells are comparable, in the latter case, there was a greater level of phagocytic activity and ROS production. This result can also be explained by the diversity of components in LEVs, including pro-inflammatory cytokines (IFNy, IL-1β, and TNFα), which can stimulate monocyte-like cells to phagocytose [33]. Additionally, the increased activity of THP-1 cells may be linked to an increase in the expression level of TLR4 (CD284) after co-culturing with LEVs derived from TNFα-activated NK cells [45,64].

It should be noted that, in contrast to THP-1 cells, the pre-incubation of PBMCs with LEVs produced via TNFα-activated NK-92 cells led to a decrease in the intensity of phagocytosis and ROS production. This result can be explained by the effect of LEVs on other cell populations of PBMCs, which could alter the effect of LEVs on monocytes. Further study is required to clarify the mechanisms through which cells of the microenvironment modulate the effects of LEVs on target cells.

## 4. Materials and Methods

### 4.1. Cell Lines

NK-92 cells (ATCC, Manassas, VA, USA), which are a model for activated NK cells [36,66,67], and THP-1 cells, representing monocytes, [51] were used. The cells were cultured in accordance with ATCC guidelines. All experiments were conducted in a humidified atmosphere at 37 °C and 5% CO_2_. Cell viability was assessed using trypan blue and propidium iodide solutions [68], and it was at least 95%. To work with EVs, all solutions, media, and fetal bovine serum (FBS) (F2442, Sigma-Aldrich, Saint Louis, MO, USA) were filtered through 0.2-micron pore filters (Sigma-Aldrich Chemical Co., Saint Louis, MO, USA) to ensure sterility [69]. The manufacturer guarantees the triple filtration of the serum through 0.1-micron membrane under aseptic conditions. Due to protocols for the isolation of EVs from biological fluids published by Gelderman, M.P., and Simak, J. [70], Ectosomes\LEVs are unstable extracellular structures; freezing and defrosting the samples lead to the destruction of ectosomes\LEVs. Therefore, in this work, we used sequentially frozen and then thawed FCS, which was further inactivated according to the standard protocol. These procedures additionally guaranteed the absence of contaminant ectosomes\LEVs from the serum. We did not perform fetal bovine serum (FBS) ultracentrifugation, as according to some researchers [71,72,73]. FBS depleted via ultracentrifugation may affect and support cell growth in a different way in comparison to regular FBS.

### 4.2. LEVs Isolation

LEVs produced via NK-92 were isolated using the method of differential centrifugation of the culture medium [23,74]. NK-92 cells were incubated at a concentration of 4 × 10^5^ cells per mL for 24 h in a volume of 40 mL of fresh medium. Some of these cells were incubated in the presence of 100 U/mL of TNFα (R&D, Santa Clara, CA, USA). Following this, LEVs were isolated from the remaining supernatant via successive centrifugations at 500× *g* (4 °C, 10 min), 9900× *g* (4 °C, 10 min), and 19,800× *g* (4 °C, 20 min).The method used allows for the isolation of LEVs with sufficient purity and a minimal loss of biological material [21,75]. Protein content in the LEVs samples was measured using the Bradford assay [76] with a NanoDrop One spectrophotometer, from Thermo Scientific (Waltham, MA, USA). The protein content in LEVs isolated from the medium of control NK-92 cells and activated with TNFα NK-92 cells was 3.3 ± 0.3 μg per 10^6^ cells and 2.8 ± 0.3 μg per 10^6^ cells, respectively. Previously, we described the phenotype ([28,34]), content ([43,77]), and effects of LEVs produced via NK-92 on target cells ([42,78]).

### 4.3. Laser Correlation Analysis

Granulometric analysis was conducted to monitor the size of isolated LEVs using a laser correlation spectrometer, Zetasizer NanoZS (Malvern Instruments, Malvern, UK). The size of LEVs isolated from the culture medium of NK-92 cells ranged from 232 to 556 nanometers, with a peak distribution at 339 nanometers, which is consistent with previously reported data [43,69,74,79]. Previously, using the methods of Laser Correlation Analysis, atomic force microscopy, electron microscopy, and flow cytofluorometry, we showed that the LEVs of NK-92 cells, which we obtain using differential centrifugation (Section 4.2), have similar results when describing the sizes [80]. Using electron microscopy, we showed the spherical symmetry of the LEVs produced by NK-92 cell line and the presence of a membrane made of a lipid bilayer [80].

### 4.4. Evaluation of the Transfer of a Fluorescent Label from LEVs Derived from NK-92 Cells to THP-1 Cells

The method described previously was utilized [42]. Prior to the experiment, the THP-1 culture medium was refreshed. THP-1 cells were seeded into the wells of a 24-well plate for suspension cultures at a concentration of 5 × 10^5^ cells per 500 μL of a complete medium for THP-1 cells supplemented with 10% FBS and incubated for 24 h. To label intracellular proteins, NK-92 cells were incubated with a solution of 5(6)-carboxy-fluorescein diacetate succinimidyl ester (CFSE) (Sigma-Aldrich Chemical Co., USA) at a concentration of 50 μM in accordance with the manufacturer’s guidelines. Intact and stained NK-92 cells were then cultured for 24 h in 75 cm^2^ flasks in 40 mL of complete culture medium at a concentration of 4 × 10⁵ cells per mL. LEVs were isolated as previously described. The LEVs were then seeded into the wells containing THP-1 cells at a concentration of 10 μg of protein per 100 μL, and co-cultured for 24 h. The cells were then washed three times with versene solution, resuspended in a Hank’s solution without Ca^2+^ and Mg^2+^, and centrifuged at 200× *g* for 10 min to remove the supernatant. The incorporation of the fluorescent form of CFSE into the membrane of THP-1 cells was assessed using a FACSCanto II flow cytometer (BD, Franklin Lakes, NJ, USA), as previously described (Supplementary Data, Figure S1) [42]. The experiments were conducted in triplicate.

### 4.5. Evaluation of the Effect of LEVs of NK-92 Cells on the THP-1 Cells Viability

THP-1 cells were prepared as we mentioned in Section 4.4. Intact and activated via 400 U/mL of TNFα NK-92 cells were cultured for 24 h in 75 cm^2^ flasks in 40 mL of complete culture medium at a concentration of 4 × 10^5^ per mL. LEVs were then isolated as described above. Following isolation, LEVs from the NK-92 cells were added to the wells containing THP-1 cells at a concentration of 5, 10, or 20 μg of protein per 100 μL. The cells were then cultured for 24 h. Then, the THP-1 cells were washed twice with Hank’s solution via centrifugation at 200× *g* for 10 min. After that, the cells were treated with a 7-amino-actinomycin D solution (7-AAD), in accordance with the manufacturer’s instructions (BD, USA). The fluorescence intensity was measured using a FACS Canto II flow cytometer (BD, USA). All experiments were conducted in triplicate, with three replicates for each concentration of LEVs.

### 4.6. Evaluation of the Effect of LEVs Derived from NK-92 Cells on Phagocytic Activity and Oxidative Burst in THP-1 Cells

THP-1 cells were prepared as we mentioned in Section 4.4 and Section 4.5. LEVs were isolated as described above and added to wells containing intact THP-1 cells at a concentration of 10 μg of protein per 100 μL. TNFα was also added to a separate group of THP-1 cells as a control for phagocytosis activation and oxidative burst. Intact cells were used as a control. After 24 h of incubation, the plate with THP-1 cells was centrifuged at 200× *g* for 10 min. Then 150 μL of the supernatant from each well was collected. The cells were then resuspended in 50 μL of medium and transferred to Eppendorf tubes. The phagocytic activity of the cells was evaluated using commercial kits (IngoFlowEx, Exbio, Vestec-Jesenice u Prahy, Czech Republic) with a BD FACSCanto II flow cytometer (BD, USA). The IngoFlowEx Kit is designed for the quantification of phagocytic activity by measuring the ingestion of fluorescently labeled (FITC) *E. coli* bacteria. The oxidative activity of the cells was evaluated using commercial kits (FagoFlowEx, Exbio, Czech Republic)with a BD FACSCanto II flow cytometer (BD, USA). The FagoFlowEx Kit is intended for the examination of phagocytic activity by measuring the respiratory (oxidative) burst after stimulation with *E. coli* bacteria in human heparinized whole blood using flow cytometry. Reactive oxygen species (ROS) production after *E. coli* incorporation via phagocytes was measured using dihydrorhodamine 123 which was then oxidized to rodamine 123 and then became fluorescent (Supplementary Data, Figures S2 and S4) Thus, the MFI of phagocytic cells is proportional to intracellular ROS activity (Figure 4). All experiments were conducted in triplicate, with three replicates for each cultivation condition.

### 4.7. Evaluation of the Effect of LEVs Derived from NK-92 Cells on Phagocytic Activity and the Realization of Oxidative Explosion via the PBMCs of Healthy Donors

Peripheral blood mononuclear cells (PBMCs) were isolated from the peripheral blood of 12 healthy donors through Ficoll density gradient centrifugation (ρ = 1.077 g/mL, Biolot, St. Petersburg, Russia), following standard procedures. The isolated PBMCs were seeded into 96-well plates at a concentration of 2 × 10^5^ cells/200 μL of complete cell culture medium. After 24 h LEVs produced by either intact or TNFα-activated NK-92 cells were added to wells at a concentration of 10 μg of protein per 100 μL. The phagocytic and oxidative activities of the PBMCs were then evaluated using commercial kits (IngoFlowEx and FagoFlowEx, Exbio, Czech Republic) and a BD FACS Canto II flow cytometer. The study was reviewed and approved by the local ethics committee of the Federal State Budgetary Scientific Institution “The Research Institute of Obstetrics, Gynecology and Reproductology named after D.O. Ott” (Protocol No. 107, dated 15 March 2021). Donors whose data were used in the work provided informed consent to their participation in the study. Subsequently, the data were de-identified and the results were analyzed.

### 4.8. Evaluation of the Effect of LEVs Derived from NK-92 Cells on the Phenotype of THP-1 Cells

THP-1 cells were prepared as we mentioned in Section 4.4 and Section 4.5. LEVs were isolated as described above and added to wells containing intact THP-1 cells at a concentration of 10 μg of protein per 100 μL and co-cultured for 24 h. THP-1 cells that were intact and activated via 400 U/mL of TNFα were used as controls. The cells were then washed three times with a versene solution, resuspended in Hank’s solution without Ca^2+^ and Mg^2+^, and centrifuged at 200× *g* for 10 min to remove the supernatant. A Fc-blocking reagent was used to block Fc-receptors according to the manufacturer’s instructions (Miltenyi Biotec, Gaithersburg, MD, USA). After incubation with LEVs, the THP-1 cells were stained with monoclonal antibodies for CD11a, CD11b, CD11c, CD18, CD54, HLA-DR, CD120a, CD14, CD36, CD284, and CD71 in accordance with the manufacturer’s instructions (BD Biosciences, San Jose, CA, USA). Isotypic antibodies were used as control for non-specific binding (BD, USA). The choice of antibodies was made based on our data, as well as the literature’s data on alterations to the phenotype of THP-1 cells after exposure to cytokines [53,81,82,83]. Additionally, we took into account the phenotype of NK-92 cells and their LEVs [28,42,74]; the data are summarized in Table 1. 

Fluorescence was measured using a BD FACSCanto II flow cytometer (BD, USA) (Supplementary Data, Figure S3). The experiments were conducted in triplicate.

### 4.9. Statistical Analysis

Statistical analysis was conducted using the GraphPad Prism 8 software. The nonparametric Mann-Whitney U test was used to compare the obtained data. The results were reported as “median (lower quartile, upper quartile)”.

## 5. Conclusions

**Figure 8:**
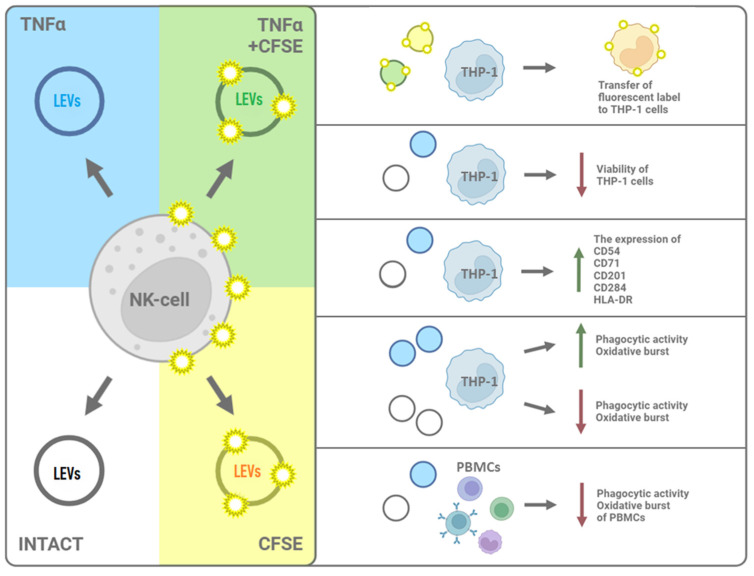
Summarizing the key findings of the study. The left side shows groups of LEVs used in the study; the right side—shows their effects on THP-1/PBMC.

In this study, we demonstrated the ability of LEVs produced via NK cells to be integrated into the membranes of THP-1 cells and influence the viability, phenotype, and functional characteristics of the cells. We supposed that the increased expression of CD54, CD71, and CD206 via THP-1 cells, as well as the appearance of CD284 and increased phagocytic and oxidative activity after a co-culture with LEVs produced through TNFα-activated NK cells, suggests that THP-1 cells acquire the characteristics of classically activated M1 macrophages [102,103]. On the contrary, in the presence of LEVs produced via intact NK cells THP-1 cells acquired the characteristics of alternatively activated macrophages (M2) [104]. The stated hypothesis requires further experimental confirmation. These cells demonstrated reduced phagocytic activity and oxidative burst intensity, as well as low expression of the M1-specific markers CD206 and CD284 [103]. However, a high number of CD54+ and CD71+ cells were observed, which is not fully consistent with the hypothesis. Therefore, further investigation is needed to determine the molecular mechanisms underlying the interaction between LEVs derived from NK cells and monocytes. The results obtained demonstrate the ability of LEVs to act as an additional component in the immunomodulatory activity of NK cells in relation to monocytes. Figure 8 represents the main results of the research.

## Figures and Tables

**Table 1:** The expression of membrane receptors in THP-1 cells, NK cells, NK-92 cells and their LEVs.

Receptor	Surface Expression				Receptor’s Function
	NK Cells	NK-92 Cells	LEVs	THP-1	
CD11a	Yes [84]	Yes [28,36]	Yes [28]	Yes [81]	Integrin
CD11b	Yes [85]	Yes [28,86]	Yes [28]	No [81]	Integrin
CD11c	Yes [85]	Yes [28,86]	Yes [28]	Yes [81]	Integrin
CD14	No [87]	No [28,36]	No data	Yes [82]	LPS receptor, coreceptor for TLR4
CD18	Yes [85]	Yes [28,88]	Yes [28]	Yes [81]	Integrin
CD36	No data	No data	No data	Yes [83]	Scavenger receptor
CD54 (ICAM-1)	Yes [89]	Yes [28,90]	Yes [28]	Yes [91]	Adhesion molecule, cell activation marker
CD71	Yes [92]	No data	No data	Yes [93]	Transferrin receptor
CD120a (TNFR1)	Yes [94]	No data	No data	Yes [53]	TNFα receptor
CD206	No [95]	No data	No data	Yes [96]	Mannose receptor
CD284 (TLR4)	Yes [97,98]	Yes [99]	No data	Yes, after activation [100]	TLR4
HLA-DR	Yes [85], after activation	No [36]	No data	Yes, after activation [101]	MHC class II cell surface receptor

## Data Availability

The data presented in this study are available upon request from the corresponding author.

## Supplementary Materials

The supporting information can be downloaded at: https://www.mdpi.com/article/10.3390/ijms25179478/s1.
