# Supplementary material for: Large Extracellular Vesicles Derived from Natural Killer Cells Affect the Functions of Monocytes
Source: Int J Mol Sci. 2024 Aug 31;25(17):9478. doi: 10.3390/ijms25179478 (PMC11395174; doi:10.3390/ijms25179478)
Supplement: Supplementary file 1 [file ijms-25-09478-s001.zip › Suppl_rev3.docx]

Table 1. The percentage (%) and mean fluorescence intensity (MFI) of THP-1 cells, incubated with LEVs derived from NK-92 cells.

|  | THP-1 | THP-1 + LEVs(int) | THP-1 + LEVs(CFSE) |
| --- | --- | --- | --- |
| % | 2,3 {2,23; 2,53 } | 3,45 {2,18; 3,9 } | 64,15 {61,03; 83,78 } ** ## |
| MFI | 476 {448; 513 } | 525 {453; 635 } | 4389 {3647; 6032 } ** ## |

THP – intact cells; THP-1+LEVS(int) – THP-1 cells cultivated in the presence of LEVs(int); THP-1+LEVs(CFSE) – THP-1 cells cultivated in the presence of LEVs(CFSE).

* - difference from THP-1; # - difference from THP-1 + LEVS(int).

Table 2. The percentage of dead THP-1 cells after their co-culturing with LEVs derived from NK-92 cells.

| THP-1(control) | THP-1+5μg LEVs(int) | THP-1+10μg LEVs(int) | THP-1+20μg LEVs(int) | THP-1+5μg LEVs(TNFα) | THP-1+10μg LEVs(TNFα) | THP-1+20μg LEVs(TNFα) |
| --- | --- | --- | --- | --- | --- | --- |
| 4,3 {2,83; 7,05 } | 5,3 {4,38; 7,43 } | 6,55 {4,23; 7,28 } | 9,2 {6,23; 13,6 } * # | 5,05 {4,68; 6,78 } | 6,65 {4,33; 6,93 } | 8,5 {6,78; 12,93 } * $ |

THP-1(control) – THP-1 cells stained with 7-AAD; 5μg/10μg/20μgLEVs(int) – THP-1 cells stained with 7-AAD after co-culture with LEVs(int) at three different concentrations (5, 10, or 20 μg of protein per 100 μl); 5μg/10μg/20μgLEVs(TNFα) – THP-1 cells stained with 7-AAD after co-culture with LEVs(TNFα) at three different concentrations (5, 10, or 20 μg of protein per 100 μl).

* - difference from THP-1(control); # - difference from THP-1+10μg LEVs(int); $ - difference from THP-1+10μg LEVs(TNFα)

Table 3. The percentage of FITC-positive THP-1 cells after phagocytosis of FITC-labeled E.coli.

| THP-1(unst) | THP-1(unst) +TNFα | THP-1(unst) +LEVs(int) | THP-1(unst) +LEVs(TNFα) | THP-1(control) | THP-1(control) +TNFα | THP-1(control) +LEVs(int) | THP-1(control) +LEVs(TNFα) | THР-1(E.coli) | THP-1(E.coli) +TNFα | THP-1(E.coli) +LEVs(int) | THP-1(E.coli) +LEVs(TNFα) |
| --- | --- | --- | --- | --- | --- | --- | --- | --- | --- | --- | --- |
| 1,45 {1,33; 2,0 } | 1,9 {1,58; 2,23 } | 2,4 {0,85; 2,53 } | 2,05 {0,85; 2,2 } | 1,45 {1,33; 2,0 } ** | 1,8 {1,55; 1,98 } | 1,8 {1,65; 2,13 } ## | 1,65 {1,08; 2,73 } | 18,25 {17,9; 18,83 } | 20,4 {18,8; 21,05 } * $$$ | 9,45 {7,5; 11,03 } *** | 22,3 {21,28; 23,73 } *** ## & |

THP-1(unst) – intact THP-1 cells; THP-1(control) – THP-1 cells stained with Tripan Blue (TB) and Propidium Iodide (PI); THP-1(E.coli) – THP-1 cells stained with TB and PI after incubation with FITC-labeled E.coli; +TNFa – THP–1 cells pre-incubated with TNFα; +LEVs(int) – THP–1 cells pre-incubated with LEVs(int); +LEVS(TNF) – THP–1 cells pre-incubated with LEVs(TNFα).

* - difference from THР-1(E.coli); # - difference from THP-1(E.coli) +LEVs(int); $ - difference from THP-1(control) +TNFα; & - difference from THP-1(control) +LEVs(TNFα)

Table 4. The mean fluorescent intensity (MFI) of THP-1 cells after phagocytosis of unstained E. coli in the presence of dihydrorhodamine 123.

| THP-1(unst) | THP-1(unst) +TNFα | THP-1(unst) +LEVs(int) | THP-1(unst) +LEVs(TNFα) | THP-1(control) | THP-1(control) +TNFα | THP-1(control) +LEVs(int) | THP-1(control) +LEVs(TNFα) | THР-1(E.coli) | THP-1(E.coli)+TNFα | THP-1(E.coli) +LEVs(int) | THP-1(E.coli) +LEVs(TNFα) |
| --- | --- | --- | --- | --- | --- | --- | --- | --- | --- | --- | --- |
| 201 {145; 206 } *** ## | 148 {108; 163 } | 155 {139; 220 } | 214 {156; 236 } | 15681 {15319; 16285 } | 16626 {16426; 17860 } $ | 14878 {12944; 16285 } && | 24181 {19865; 24781 } %% | 15728 {15435; 17679 } | 17668 {16930; 19726 } ** $$$ | 12632 {11333; 13126 } *** && | 22404 {20824; 24713 } *** @@@ %%% |

THP-1(unst) – intact THP-1 cells; THP(control) – THP-1 cells treated with phorbol-12-myristate-13-acetate (PMA); THP(E. coli) – THP-1 cells that have undergone oxidative explosion after activation with FITC-labeled E.coli; +TNFa – THP–1 cells pre-incubated with TNFα; +LEVs(int) – THP–1 cells pre-incubated with LEVs(int); +LEVs(TNFα) – THP–1 cells pre-incubated with LEVs(TNFα).

* - difference from THР-1(E.coli); # - difference from THP-1(control) +LEVs(int); $ - difference from THP-1(unst) +TNFα; & - difference from THP-1(unst) +LEVs(int); @ - difference from THP-1(E.coli) +LEVs(int); % - difference from THP-1(unst) +LEVs(TNFα).

Table 5. The percentage of THP-1 cells expressing receptors in the presence of LEVs(int) or LEVs(TNFα).

|  | IC | THP-1(int) | THP-1+TNFα | THP-1+LEVs(int) | THP-1+LEVs(TNFα) |
| --- | --- | --- | --- | --- | --- |
| HLA-DR | 1,5 {1,2; 2,3 } | 0,9 {0,65; 1,15 } | 1,0 {0,8; 2,3 } | 2,4 {2,15; 2,65 } ** ### | 5,8 {4,5; 5,9 } *** $$$ |
| CD54 | 1,6 {1,1; 2,3 } | 40,7 {29,75; 43,1 } *** | 98,4 {97,5; 99,1 } *** ### | 67,5 {51,4; 69,95 } *** ### | 92,7 {89,65; 94,8 } *** $$$ |
| CD71 | 1,8 {1,25; 2,35 } | 3,7 {3,2; 4,1 } *** | 7,8 {7,1; 12,7 } *** ### | 9,8 {8,25; 11,35 } *** ### | 18,2 {16,9; 31,4 } *** $$$ |
| CD284 | 2,0 {1,65; 2,4 } | 0,6 {0,1; 1,15 } | 5,5 {4,6; 6,0 } * ### | 4,4 {1,25; 10,45 } ### | 11,3 {8,3; 13,8 } *** $ |
| CD14 | 2,1 {1,3; 2,6 } | 89,5 {60,2; 93,1 } *** | 93,7 {75,7; 97,0 } *** | 92,7 {58,6; 95,75 } *** | 95,3 {67,95; 96,0 } *** |
| CD36 | 2,15 {1,93; 2,38 } | 54,9 {25,8; 100,0 } *** | 65,8 {41,05; 99,95 } *** | 33,6 {29,45; 99,95 } *** | 36,0 {25,55; 100,0 } *** |
| CD11a | 2,2 {1,55; 2,2 } | 99,5 {97,6; 100,0 } *** | 99,1 {98,15; 100,0 } *** | 99,1 {97,9; 100,0 } *** | 99,1 {98,3; 100,0 } *** |
| CD11b | 2,3 {2,03; 2,75 } | 99,8 {98,35; 99,9 } *** | 99,8 {99,4; 99,95 } *** | 99,2 {97,85; 100,0 } *** | 99,3 {96,85; 100,0 } *** |
| CD11c | 2,05 {1,58; 2,45 } | 19,2 {8,8; 21,35 } *** | 23,5 {19,4; 100,0 } *** | 58,45 {16,5; 100,0 } *** | 61,3 {19,83; 100,0 } *** |
| CD206 | 2,1 {1,2; 2,55 } | 3,3 {2,45; 4,7 } ** | 17,2 {13,8; 23,8 } *** ### | 4,9 {4,05; 6,2 } *** # | 7,7 {6,05; 9,5 } *** $ |
| CD120a | 2,3 {1,25; 2,7 } | 35,1 {28,75; 50,4 } *** | 64,4 {48,8; 67,8 } *** ## | 42,5 {31,75; 47,65 } *** | 49,6 {22,7; 55,7 } *** |
| CD18 | 2,3 {2,2; 2,8 } | 96,6 {94,4; 99,4 } *** | 98,4 {98,1; 99,35 } *** | 98, {97,6; 99,8 } *** | 98,6 {97,4; 99,55 } *** |

IC – isotypic control; THP-1(int) – intact THP-1 cells; THP-1+TNFα – THP-1 cells pre-incubated with TNFα; THP-1+LEVs(int) – THP-1 cells pre-incubated with LEVs(int); THP-1+LEVs(TNFα) – THP-1 cells pre-incubated with LEVs(TNFα).

* - difference from IC; # - difference from THP-1(int); $ - difference from THP-1+LEVs(int).

Table 6. The mean fluorescence intensity (MFI) of THP-1 cells expressing receptors in the presence of LEVs(int) or LEVs(TNFα).

|  | IC | THP-1(int) | THP-1+TNFα | THP-1+LEVs(int) | THP-1+LEVs(TNFα) |
| --- | --- | --- | --- | --- | --- |
| HLA-DR | 2480,0 {2244,0; 2543,0 } | 4213,0 {3701,0; 5224,0 } *** | 4341,0 {3904,0; 4486,0 } *** | 4550,0 {4211,0; 5292,0 } *** | 5035,0 {4485,0; 5242,0 } *** |
| CD54 | 8306 {7742; 8582 } | 8936 {8851; 9807 } ** | 40679 {39076; 41743 } *** ### | 13615 {12931; 13948 } *** ### | 25641 {24980; 30326 } *** $$$ |
| CD71 | 6954 {6882; 7500 } | 7737 {7599; 8628 } ** | 7887 {7361; 8289 } * | 8175 {7979; 8767 } *** | 9186 {8672; 9453 } *** $$ |
| CD284 | 8053 {7971; 8467 } | 10993 {9987; 13558 } *** | 9115 {8771; 12540 } ** | 12890 {8698; 14237 } *** | 9477 {9307; 10206 } *** |
| CD14 | 615 {519; 625 } | 803 {719; 850 } *** | 886 {880; 1197 } *** ### | 782 {741; 1057 } *** | 848 {784; 1087 } *** |
| CD36 | 1711 {375; 2048 } | 2577 {1486; 2961 } * | 2719 {1778; 3062 } * | 2700 {1486; 2899 } * | 2736 {1619; 3020 } * |
| CD11a | 28765 {670; 29514 } | 131163 {114622; 158670 } *** | 126617 {119137; 141621 } *** | 136309 {118329; 156294 } *** | 137208 {122220; 147668 } *** |
| CD11b | 2581 {880; 3489 } | 5931 {5671; 7955 } *** | 7043 {6336; 7316 } *** | 6323 {5881; 6630 } *** | 6173 {6143; 6951 } *** |
| CD11c | 8066 {272; 10167 } | 8828 {5895; 12490 } | 9158 {6680; 11591 } | 9088 {6208; 11715 } | 9374 {6501; 11441 } |
| CD206 | 7997 {6938; 8602 } | 8161 {7108; 8640 } | 8902 {8647; 9394 } ** ## | 8813 {7146; 9101 } | 8834 {8367; 9592 } * |
| CD120a | 2523 {2217; 2704 } | 2953 {2897; 3267 } ** | 3289 {3075; 3613 } ** | 3123 {2886; 3401 } ** | 3201 {3066; 3398 } ** |
| CD18 | 2127 {1723; 2286 } | 4933 {4496; 7643 } *** | 5755 {5453; 8659 } *** | 5885 {4967; 10264 } *** | 5590 {5324; 8027 } *** |

IC – isotypic control; THP(int) – intact THP-1 cells; THP+TNFα – THP-1 cells pre-incubated with TNFα; THP-1+LEVs – THP-1 cells pre-incubated with LEVs(int); THP-1+LEVs(TNFα) – THP-1 cells pre-incubated with LEVs(TNFα).

* - difference from IC; # - difference from THP-1(int); $ - difference from THP-1+LEVs(int).

Table 7. The percentage of PBMCs that phagocytized FITC-labeled E. coli in the presence of LEVs(int) or LEVs(TNFα).

| РВМСs(unst) | РВМСs(unst) +LEVs(int) | РВМСs(unst) +LEVs(TNFα) | РВМСs(control) | РВМС(control) +LEVs(int) | РВМСs(control) +LEVs(TNFα) | РВМСs(E.coli) | РВМСs(E.coli) +LEVs(int) | РВМСs(E.coli) +LEVs(TNFα) |
| --- | --- | --- | --- | --- | --- | --- | --- | --- |
| 2,05 {1,8; 2,05 } | 2,3 {1,3; 2,3 } | 1,8 {1,05; 1,8 } | 1,0{0,65; 1,0 } *** | 2,1 {1,45; 2,1 } | 1,3 {0,9; 1,3 } | 35,1 {23,75; 35,1 } | 14,15 {9,45; 14,15 } *** ### | 14,8 {10,2; 14,8 } *** $$$ |

PBMCs(unst) - oxidative burst of intact PBMCs; PBMCs(control) - THP-1 cells stained with Tripan Blue (TB) and Propidium Iodide (PI); PBMCs(E. coli) – baseline level of oxidative burst of PBMCs stimulated by FITC-labeled E. coli; + LEVs(int) – PBMCs treated with LEVs(int); + LEVs(TNFα) – PBMCs treated with LEVs(TNFα).

* - difference from РВМСs(E.coli); # - difference from РВМС(control)+LEVs(int); $ - difference from РВМСs(control)+LEVs(TNFα).

Table 8. The mean fluorescence intensity (MFI) of PBMCs that phagocytized FITC-labeled E. coli in the presence of LEVs(int) or LEVs(TNFα).

| РВМСs(unst) | РВМСs(unst) +LEVs(int) | РВМСs(unst) +LEVs(TNFα) | РВМСs(E.coli) | РВМСs(E.coli) +PMA | РВМСs(E.coli) +LEVs(int) | РВМСs(E.coli) +LEVs(TNFα) |
| --- | --- | --- | --- | --- | --- | --- |
| 2562 {1418; 2990 } *** | 2008 {1542; 2390 } | 1837 {1238; 2271 } | 27914 {16718; 31391 } | 33189 {32508; 39241 } *** | 13073 {11624; 14914 } *** ### | 15031 {12638; 16410 } ** $$$ |

PBMCs(unst) - oxidative burst of intact PBMCs; PBMCs(E. coli) – baseline level of oxidative burst of PBMCs stimulated by FITC-labeled E. coli; +PMA – oxidative burst in the presence of PMA (phorbol-12-myristate-13-acetate); + LEVs(int) – PBMCs treated with LEVs(int); + LEVs(TNFα) – PBMCs treated with LEVs(TNFα).

* - difference from РВМСs(E.coli); # - difference from РВМС(unst)+LEVs(int); $ - difference from РВМСs(unst)+LEVs(TNFα).


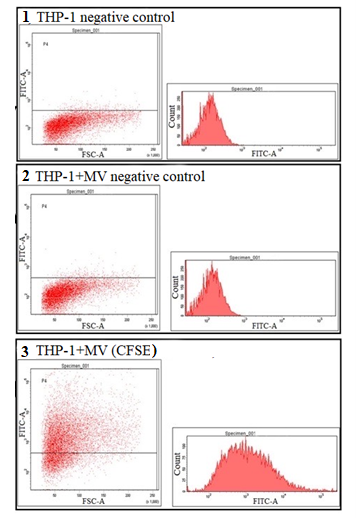


Supplementary figure 1. Gating strategy used in experiment “Evaluation of the transfer of a fluorescent label from LEVs derived from NK-92 cells to THP-1 cells”. 1 – autofluorescence of THP-1 cells (FITC), 2 – THP-1 cells after incubation with LEVs derived by intact NK-92 cells, 3 – THP-1 cells after incubation with LEVs derived by CFSE-stained NK-92 cells.


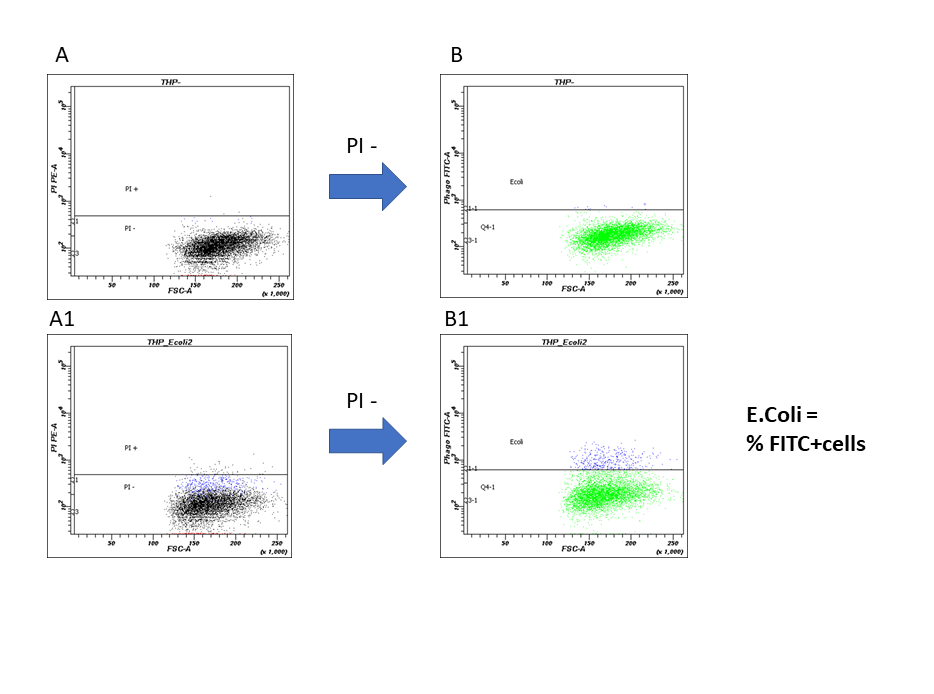


Supplementary figure 2. Gating strategy used in experiment “Evaluation of the effect of LEVs derived from NK-92 cells on phagocytic activity of THP-1 cells”.
A,A1 – THP-1 cells positve or negatative for PI staining (PE), В, В1 – THP-1 cells positive or negative for phagocytosinf of E.coli (CFSE stained) (FITC).


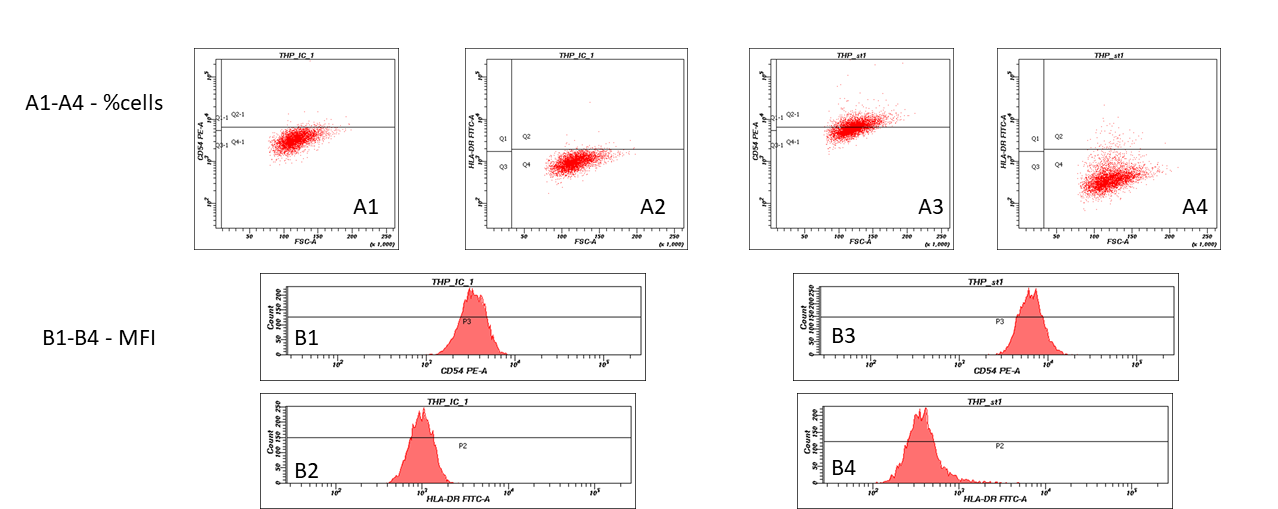


Supplementary figure 3. Gating strategy used in experiment “Evaluation of the effect of LEVs derived from NK-92 cells on the phenotype of THP-1 cells”. ). Left side (A1, A2, B1, B2) shows THP-1 cells treated with isotypical controls, right side (A3, A4, B3, B4) – THP-1 cells stained with antibodies. Gates are set by isotypical controls. A1-A4 show percent of cells % of THP-1 cells which express CD54/HLA-DR respectevely (these molecules shown as an example). B1-B4 – MFI CD54/HLA-DR respectevely.


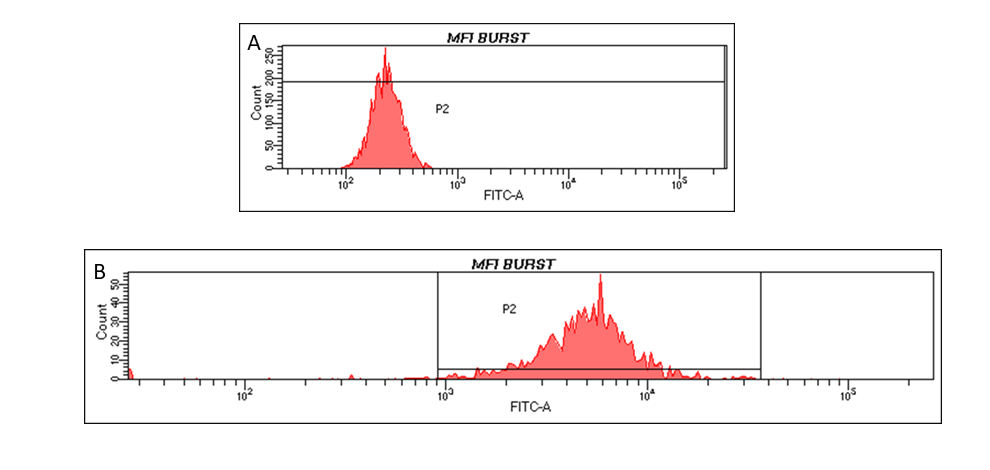


Supplementary figure 4. Gating strategy used in experiment “Evaluation of the effect of LEVs derived from NK-92 cells on oxidative burst in THP-1 cells”. А – MFI (FITC) of THP-1 cells before phagocytosis of unstained E. coli (before the activation) in the presence of dihydrorhodamine 123 (P2), В – MFI (FITC) of THP-1 cells after phagocytosis of unstained E. coli (after the activation) in the presence of dihydrorhodamine 123 (P2).
