# Supplementary figures and images for: Large Extracellular Vesicles Derived from Natural Killer Cells Affect the Functions of Monocytes
Source: Int J Mol Sci. 2024 Aug 31;25(17):9478. doi: 10.3390/ijms25179478 (PMC11395174; doi:10.3390/ijms25179478)

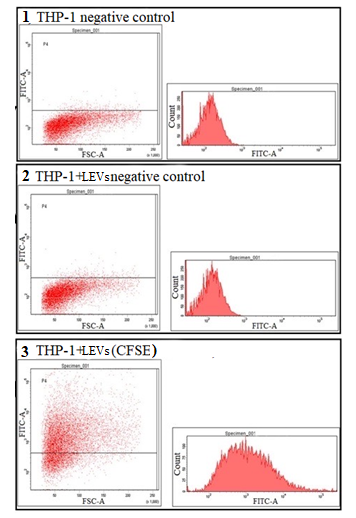

Supplement: Supplementary file 1 [file ijms-25-09478-s001.zip › SF1.tif]

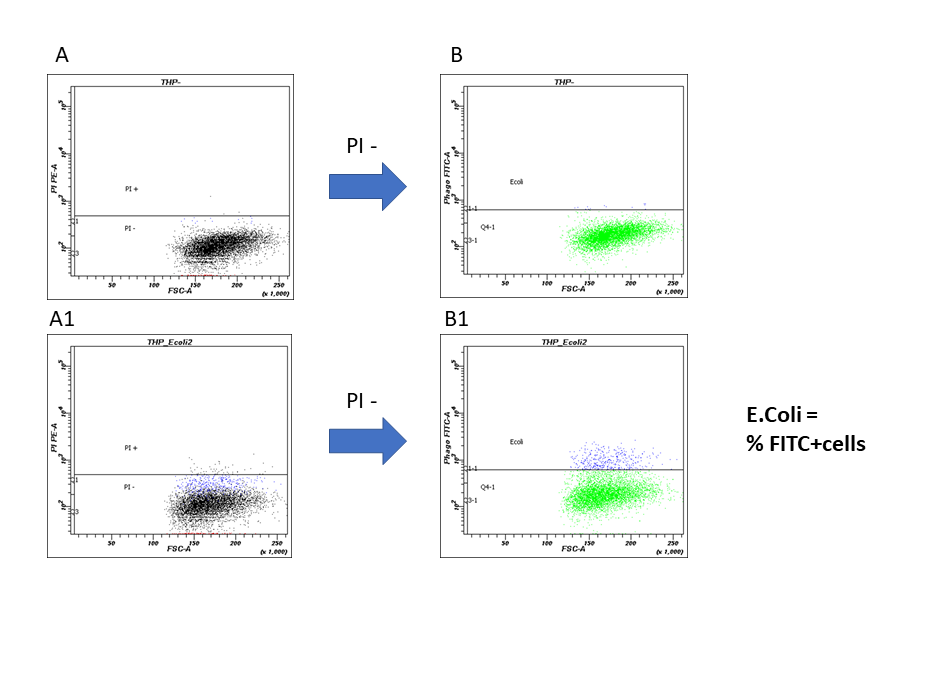

Supplement: Supplementary file 1 [file ijms-25-09478-s001.zip › SF2.tif]

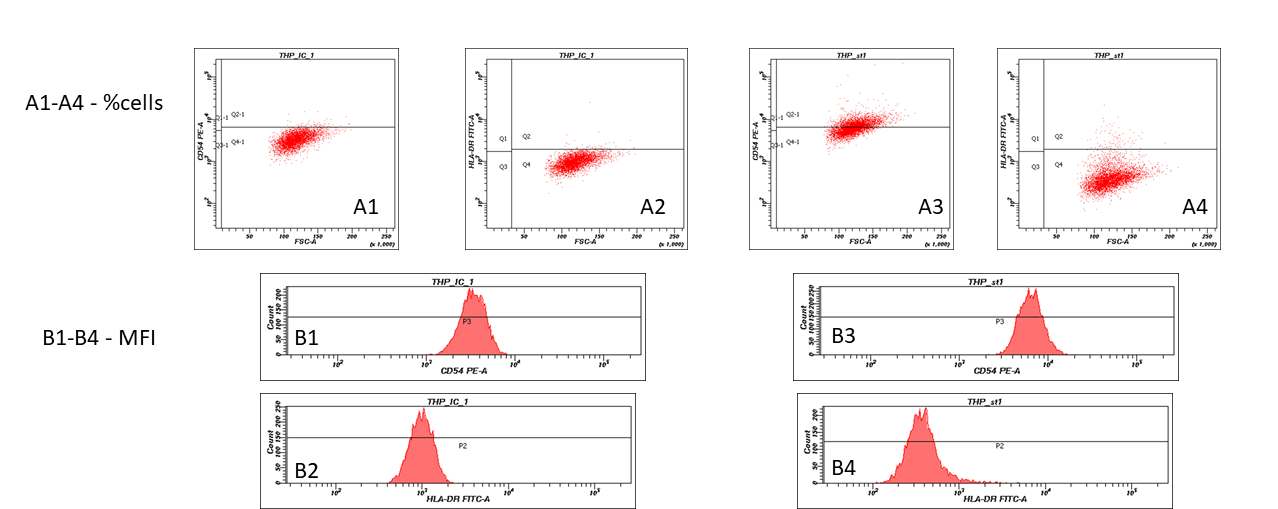

Supplement: Supplementary file 1 [file ijms-25-09478-s001.zip › SF3.tif]

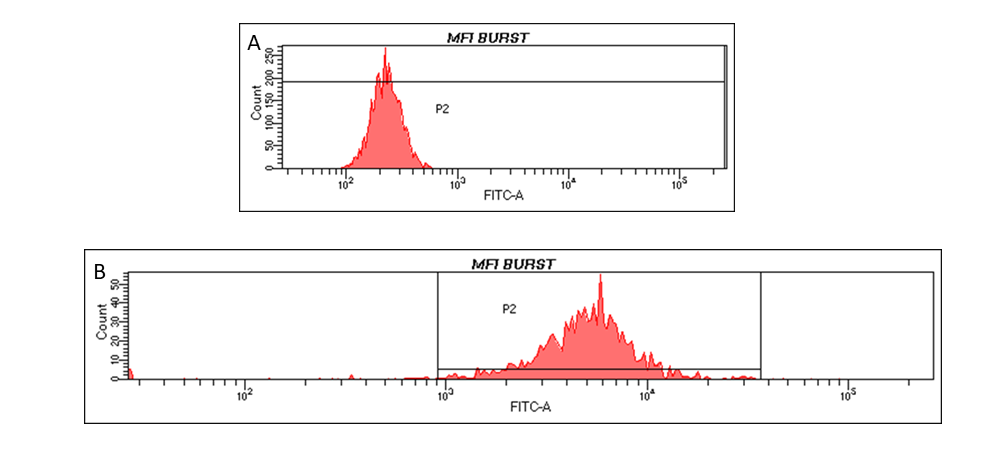

Supplement: Supplementary file 1 [file ijms-25-09478-s001.zip › SF4.tif]
